# Nature-Inspired Trojan Materials as Invisible Enablers of Advanced Humidity Sensors

**DOI:** 10.3390/ma19112402

**Published:** 2026-06-04

**Authors:** Daniela S. Oliveira, Elizabeth S. Vieira, Gabriela V. Martins, Joana J. Costa, Rafaela M. Meira, Carlos A. Ramos, Daniela Campanhã, Gina Vilão, Joaquim Alves, P. Filipe Santos, Felismina T. C. Moreira

**Affiliations:** 1 CIETI—LabRISE, ISEP, Polytechnic of Porto, Rua Dr. António Bernardino de Almeida, 431, 4249-015 Porto, Portugal; ddsol@isep.ipp.pt (D.S.O.); jjdca@isep.ipp.pt (J.J.C.); jaa@isep.ipp.pt (J.A.); 2 Prudêncio Impermeabilizações, Parque Industrial Sete Fontes, R. Dos Pedreiros 14, 4710-553 Braga, Portugal; elizabeth@prudencio.pt (E.S.V.); filipe@prudencio.pt (P.F.S.); 3 CENTITVC—Centro Nanotecnologia e Materiais TFI, Quinta da Maia—Rua Fernando Mesquita, N° 2785, 4760-034 Vila Nova de Famalicão, Portugal; rmeira@centi.pt (R.M.M.); dcampanha@centi.pt (D.C.); 4 CIETI—NBIN, ISEP, Polytechnic of Porto, Rua Dr. António Bernardino de Almeida, 431, 4249-015 Porto, Portugal; car@isep.ipp.pt (C.A.R.); gmr@isep.ipp.pt (G.V.)

**Keywords:** humidity sensors, nature-inspired materials, Trojan materials, bioinspired nanomaterials, sensor performance enhancement

## Abstract

Nature offers a robust conceptual framework for designing next-generation adaptive, multifunctional sensing systems. Also, in sensing systems, Trojan materials add a functional dimension to the microstructure, enabling the development of high-performance humidity sensors without interfering with their macrostructure. Thus, based on a brief overview of how inspiration from plants, animals, and membranes can be used to engineer high-performance platforms for environmental humidity monitoring, combined with the functional dimension of Trojan materials, this review presents a critical framework detailing the key developments in the main categories of self-sensing materials within the scope of humidity sensors. The review addresses electronically and ionically conductive polymers, polymer composites with dispersed active fillers, and hydrogel-based or other water-compatible systems. Additionally, commercially available sensors are described, and the main challenges and future directions are identified.

## 1. Introduction

In recent decades, rapid industrialization and urban growth have driven technological and economic advances, but have also triggered crises such as climate change, environmental degradation, biodiversity loss, and resource depletion [1]. In response, global initiatives such as the Global Biodiversity Framework (GBF) coordinate strategies to support biodiversity conservation and climate mitigation (Convention on Biological Diversity, 2022). Active implementation requires new scientific evidence and innovative technologies. In environmental monitoring, humidity sensors are particularly valuable, as they enable precise monitoring of humidity, thereby supporting early detection of ecological disorders, providing guidance for habitat management and climate impact evaluation, as well as supporting public health initiatives [1,2,3]. In humidity sensors, the concept of Trojan materials involves embedding functional materials within the sensing layer to unobtrusively enhance water adsorption, transport, or signal transduction, thereby improving performance without altering the sensor’s apparent geometry, flexibility, or mechanical durability [4,5].

Typical electrochemical humidity sensors consist of a substrate, electrodes, and a humidity-sensitive layer. Their operation relies on the reversible adsorption and desorption of water molecules by the sensing material in response to changes in ambient humidity [6]. These interactions produce measurable electrical variations, including changes in resistance, capacitance, or impedance, which are afterwards translated into humidity measurements [7,8]. A wide-ranging variety of materials has been studied for this purpose, including carbon-based nanomaterials, polymers, metal oxides, and naturally derived nanofibers [9]. Optimizing the microstructure of the sensing layer is crucial to facilitate absorption and desorption, enhancing both the response and recovery times critical for real-time monitoring [10,11].

Within this framework, Trojan materials act as hidden enhancers embedded in the active layer of the sensor. Their effects are largely invisible at the macroscopic level but can modulate electrical, ionic, or dielectric properties internally. In contrast to conventional modifications such as coatings or structural changes, Trojan materials work at the micro- or nanoscale, providing advances without changing the device’s external appearance [12]. Common mechanisms include: (i) Amplified water adsorption through hydrophilic nanoparticles or porous [13]; (ii) Enhanced ionic or protonic transport within polymeric matrices [14,15]; and (iii) Dielectric modulation to improve capacitive, resistive or impedimetric signals [16,17]. This approach could be inspired by biological systems such as camouflage or immune evasion, where critical functions are hidden yet remain highly effective [18].

In the literature, there are numerous review articles regarding humidity sensors [10,13,19]. However, there is a gap regarding a synthesis that highlights the convergence of three domains—biomimetics, Trojan materials, and sensors—that lead to the design and development of smart and sustainable humidity-sensing materials. Therefore, this review aims to focus on the integration of these three domains, providing a critical framework. Biomimetics provides innovative solutions for water management: leaf-inspired hierarchical architectures enhance water adsorption, transport, and sensor performance [18,19,20], while artificial spider silks enable controlled droplet transport and the development of new water-collection materials [9]. By replicating these structures, researchers have designed surfaces that capture and transport moisture with energy efficiency and response speed beyond those achievable by traditional synthetic methods. The integration of Trojan materials adds a functional dimension, enabling the development of humidity sensors in which water uptake alters the material’s internal architecture to signal changes without compromising the macroscopic structure of the sensors. This interfacial and confined charge mediated functionality can support the design of passive, low cost, and potentially self-powered devices, although these characteristics depend strongly on material selection and system integration. In practice, the self-powered operation of humidity sensors is typically realized through coupling with triboelectric, piezoelectric, or photovoltaic components that convert environmental stimuli into usable electrical signals [21,22]. Similarly, polymer and biopolymer-based Trojan systems contribute to sustainability and cost reduction due to their abundance and low temperature processability. However, nanocomposite architectures involving compound carbon nanotube (CNTs), graphene, or metal–organic framework (MOFs) may still present scalability and fabrication complexity challenges [23,24,25]. This overview is important because the integration of biomimetics and Trojan materials into humidity sensors allows overcoming the limitations of conventional sensors (such as low sensitivity or mechanical fragility) through an intelligent design approach inspired by nature.

Beyond the Introduction, this review includes seven additional sections: Section 2 catalogs bioinspired water transport strategies applicable to humidity sensors. Section 3 presents the critical framework and examples demonstrating how certain materials used in humidity sensors, by acting as Trojan materials, can revolutionize materials science. Section 4 discusses humidity sensing mechanisms applied to different classes of Trojan materials, describing the materials used, their primary mechanisms, and sensor performance. Section 5 covers and discusses classes of bioinspired self-sensing materials with Trojan materials. Section 6 reviews commercially available humidity sensors. Section 7 outlines the main challenges and future directions in humidity sensing, and Section 8 provides the main conclusions.

## 2. Nature-Inspired Materials: Concepts and Mechanisms

Biomimetics bridges different disciplines by studying and applying nature’s structures, processes, and strategies to advance solutions in science and engineering domains [18].

**Figure 1:**
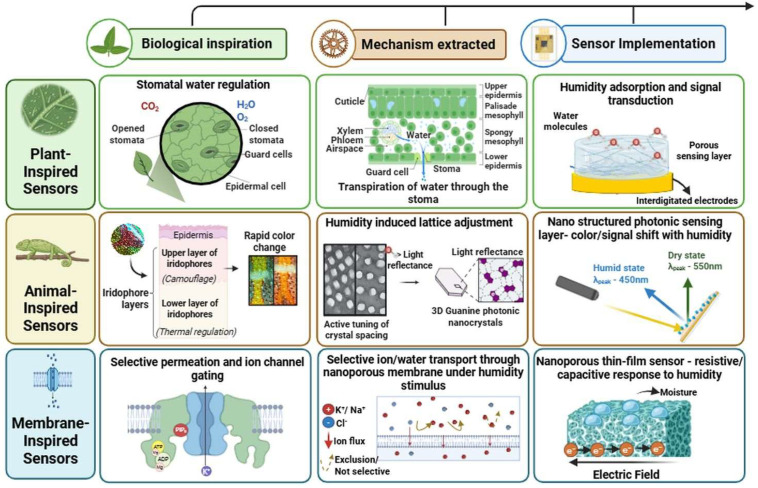
Bio-inspired strategies for humidity sensing: from natural mechanisms to sensor design. Created in Biorender. Daniela Oliveira. (2026) https://app.biorender.com/illustrations/6a04a25f0cbf9e644e247afb.

The objective is to arise strategic principles from nature rather than imitate its forms: understanding how organisms overcome challenges and converting those mechanisms into efficient, nature-inspired applications and devices [26]. Lotus leaves stay spotless through extreme water repellence (super-hydrophobicity) [27], dragonfly wings annihilate bacteria with forests of nanoscale pillars [28] (Figure 1). Cephalopods dynamically reconfigure skin color in milliseconds [29] while pangolins fuse hard armor with flexible joints to combine protection and agility [30]. Together, these systems show that biology typically achieves multifunctionality, adhesion, sensing, antifouling, and mechanical durability at extremely low energy costs. Comparative studies demonstrate recurring hierarchical architectures, graded complexity, and stimuli-responsive materials that can be directly applied to next-generation sensors [31]. Nature works on several levels: form, structure, processes, manufacturing, and ecosystem circularity [18].

Overall, structural principles shape functional design; process strategies enable production with less energy and pollution; and ecosystem thinking helps create sensors suitable for sustainable, circular systems. In contrast to conventional engineering, which typically assumes abundant energy and materials, biological strategies reliably achieve rich multifunctionality with minimal resources and a much smaller environmental footprint [32].

### 2.1. Plant-Inspired Materials

Plant-inspired humidity sensors mimic natural water management mechanisms such as selective absorption and capillary-driven transport in leaf cuticles and root hairs. These biological systems allow effective humidity capture, directional transport, and regulation, inspiring advanced sensing platforms [33]. Pinecone-inspired hygroscopic systems use anisotropic swelling of cellulose-based materials for reversible actuation and sensing over 20–90% relative humidity (RH), with 10–60 s response times [34].

More recently, leaf-inspired hierarchical architectures have been shown to enhance water adsorption, transport, and overall sensor performance. Deng et al. developed a leaf-inspired self-powered humidity sensor that mimics vein networks and stomatal distribution for capillary-driven water transport. The device exhibits response and recovery times under 5 s and high sensitivity over 20–95% RH, outperforming conventional hygroscopic systems. Its self-powered design also makes it appropriate for wearable and remote sensing applications [20]. Likewise, Li et al. reported a bioinspired leaf vein-based humidity sensor using a hygroscopic polymer, in which the vein-like microstructure improves rapid humidity diffusion and signal amplification. This sensor is sensitive, particularly at low humidity levels (<30% RH), with response times of approximately 2–10 s and good repeatability over multiple cycles [35].

Cellulose-based sensors are inspired by plant systems, where cellulose is the main structural component for water interaction and transport. Its abundant hydroxyl groups strongly adsorb moisture through hydrogen bonding, and its porous, fibrous network promotes efficient water diffusion, similar to capillary flow in plants. Humidity-induced swelling and deswelling of cellulose produce measurable electrical changes, forming the basis of its sensing mechanism. These properties make cellulose a bio-derived material that translates plant water management strategies into sustainable, high-performance humidity sensors [36,37]. Pure and chemically modified cellulose-based structures have also been extensively studied. A representative example is a flexible humidity sensor made from chemically functionalized cellulose paper, which shows stable sensing characteristics and high mechanical compliance, enabling potential use in environmental monitoring and wearable electronic platforms [38]. However, compared to composite architectures, such cellulose-based sensors generally operate over a more limited relative humidity range and exhibit reduced long-term stability. This limitation primarily results from the inherent tendency of cellulose to swell and undergo physicochemical degradation during prolonged exposure to high humidity conditions [38]. To address these limitations, cellulose has been combined with conductive polymers such as polyaniline. A resistive humidity sensor made from cellulose/polyaniline composite materials shows improved electrical responsiveness and increased sensitivity to environmental changes, especially regarding ambient humidity levels relevant to industrial and agricultural monitoring applications [39]. Similarly, inorganic–organic hybrids such as cellulose/TiO_2_ sensors exploit the hydrophilicity and large surface area of cellulose, along with the chemical stability of metal oxides, resulting in reliable operation across a wide humidity range and improved durability in environmental applications [40]. Overall, these studies show that cellulose-based humidity sensors can cover broad operating ranges, typically from about 5% to 100% RH, depending on composition, while durability is significantly enhanced in hybrid systems that combine cellulose with conductive polymers, carbon nanomaterials, or metal oxides.

Hydrogel-based sensors, inspired by plant tissues, offer high sensitivity across 10–100% RH due to their swelling behavior, though their response times (5–30 s) and long-term stability may be limited by dehydration [41].

Recent advances show that integrating plant-inspired features such as vein networks, cuticle-like selective absorption, and capillary-driven transport significantly enhances sensor performance. These strategies result in faster response times (<5 s), improved sensitivity at low relative humidity, and greater stability, making plant-inspired humidity sensors promising candidates for next-generation wearable, environmental, and agricultural monitoring systems.

Humidity sensors inspired by leaf venation and stomatal architectures exhibit high sensitivity and rapid response dynamics, primarily due to their hierarchically organized microchannel networks, and can achieve improved environmental sustainability when fabricated from cellulose-based or hydrogel-based materials. Such bioinspired configurations enable spatially resolved, long-term humidity mapping across both natural ecosystems and the built environment. Nevertheless, several limitations persist, including the mechanical and chemical fragility of the constituent materials under prolonged moisture exposure, hysteresis caused by residual water trapped within microchannels, sluggish recovery at elevated relative humidity (RH), and the substantial fabrication complexity associated with faithfully reproducing microscopic biological structures. The same microchannels that provide efficient capillary transport also promote water retention, resulting in pronounced hysteresis and slow desorption, particularly near saturation, where sensor recovery can be markedly delayed. Furthermore, accurate emulation of leaf vein and stomatal geometries typically requires advanced microfabrication techniques or bio-replication processes, thereby increasing production costs and impeding large-scale, economically viable manufacturing.

Cellulose-based hydrogels, with their porous, hydrophilic networks, enable spontaneous moisture uptake and release, allowing passive, energy-free humidity sensing through humidity-dependent ionic conductivity and dielectric permittivity. They provide biodegradability, mechanical flexibility, and high protonic conductivity via extended hydrogen-bond networks, but face challenges such as structural instability during cycling, moisture-induced softening and mechanical degradation, and hysteresis from slow water desorption. Reinforcing cellulose hydrogels with nanofillers (e.g., graphene oxide, MXenes, nanocellulose fibrils) and incorporating interpenetrating polymer networks improves mechanical robustness, environmental durability, and long-term sensing stability [42].

### 2.2. Animal-Inspired Materials

Recent advances in materials science and sensing increasingly use bioinspired strategies for fluid control, environmental monitoring and intelligent interfaces. Natural systems provide efficient solutions through micro- and nanoscale surface structures that regulate wettability and direct water transport. Biological surfaces such as insect wings and animal skins use hierarchical textures for water channeling, condensation, and self-cleaning, offering key design principles for engineered materials [43]. The Namib Desert beetle, with its patterned hydrophilic and hydrophobic regions that promote gravity-driven water condensation and transport, also inspires synthetic surfaces with controlled wettability for water harvesting [44,45,46]. Spider silk uniquely collects and transports water through spindle-knots and joints that generate surface energy and curvature gradients, driving directional microdroplet motion [47]. Artificial spider silks replicate this effect, enabling controlled droplet transport and new water collection materials [48,49].

Another study reports a biocompatible humidity sensor that uses natural inner eggshell membrane (IESM) as both the substrate and active layer, leveraging its multilayer cross-linked fiber structure. Two sizes of inkjet-printed interdigital electrodes enable capacitive and impedance-based humidity detection from 0% to 90% RH. The sensor exhibits a fast response (≈2 s) and recovery (<10 s), depending on electrode size, and stable operation at 1 kHz and 10 kHz, demonstrating the effectiveness of the porous structure for moisture sensing. The biodegradable IESM provides a sustainable, practical approach to eco-friendly, high-performance humidity sensors for everyday use [50].

Inspired by the water collection and transport capabilities of spider silk structures, a sensitive fiber humidity sensor is created by surface modification of profiled fibers with poly(3,4-ethylenedioxythiophene): poly(styrenesulfonate) (PEDOT:PSS). The fiber humidity sensor exhibits a quick response (4 s), swift recovery (29 s), and a wide operating range (6–85% RH). This performance results from the moisture absorption benefits of the bionic groove structure, the weak hydrophilicity of the profiled fibers, and the rapid moisture adsorption and desorption capacity, as well as the structural stability under bending deformation provided by the continuous lamellar PEDOT:PSS [51].

Beyond structural bioinspiration, biological sensing has also driven the development of intelligent and adaptive systems. For example, bee-inspired multisensory integration has led to neuromorphic platforms that fuse humidity and visual inputs using spiking neuron models, enabling efficient environmental perception and tasks such as authenticity recognition [43]. Similarly, camels’ ability to detect moisture in arid environments has inspired durable neuromorphic humidity sensors that locate water sources by sensing humidity gradients, offering promise for environmental monitoring and autonomous navigation [52]. Other strategies focus on optical and wearable sensing: chameleon-inspired colorimetric sensors provide real-time humidity detection through structural color changes, enabling intuitive sensing without complex electronics [46], while skin-conformal, breathable humidity sensors enable continuous monitoring of respiration and perspiration for emotional state recognition and non-contact human–machine interfaces [53].

Insect wings and animal skins are promising models for humidity-adaptive surfaces because their hierarchical micro- and nano-textures naturally control water condensation, transport, and removal. Their capillary gradients guide droplets, while hydrophobic microstructures provide self-cleaning and antifouling functions. These designs can also enable condensation-driven cooling and moisture collection for low-power, sustainable sensing materials. However, transferring these complex structures to biomimetic materials is challenging. Replicating multiscale textures requires expensive nanofabrication, and artificial replicas often lack the elasticity and durability of natural tissues, degrading under repeated wetting or UV exposure. Integrating textured surfaces with flexible or electronic substrates is also technically difficult.

Together, these studies reveal a convergence of bioinspiration, advanced materials, and intelligent systems, demonstrating that nature-derived structural design, functional materials, and sensory capabilities can produce devices with improved performance, adaptability, and multifunctionality across diverse applications.

### 2.3. Membrane-Inspired Systems

Membrane-inspired humidity sensors are a versatile technology for environmental monitoring, employing biomimetic design and advanced materials to achieve high sensitivity, flexibility, and fast response. They emulate natural membranes that selectively interact with water molecules, swell, or change electrical properties with humidity, enabling precise detection of relative humidity in diverse settings. These biomimetic sensors are suitable for wearable electronics, environmental monitoring, and soft robotics. Wang et al. report a flexible humidity sensor based on an MXene/bamboo cellulose fiber (BCF) aerogel, fabricated by vacuum filtration and freeze-drying to create a porous, moisture-interactive structure. Its sensing mechanism relies on the hygroscopic expansion of BCFs, which changes the spacing between conductive MXene layers and thus the resistance. The sensor exhibits a sensitivity of 2.46% per %RH, response and recovery times of 260 s and 282 s, respectively, and stable performance over repeated cycles and prolonged ambient exposure [54].

Materials are crucial to the performance of these sensors. Polymeric membranes, such as polyvinyl alcohol (PVA) and polyethylene oxide (PEO), are widely used for their hygroscopic properties and mechanical flexibility. A low-cost, high-performance humidity sensor based on a PEO/PVA polymer composite has been specifically designed for temperature-independent operation. By fabricating sensors with different weight ratios, the optimized composition (40:60% PEO/PVA) demonstrates stable and reliable performance across a humidity range of 0–60% RH. The sensing mechanism relies on impedance variation, with fast response and recovery times of 9 s and 16 s, respectively. The sensor maintains consistent performance despite temperature fluctuations and demonstrates excellent long-term stability over 90 days [55].

A highly compliant porous ionic membrane (PIM) humidity sensor is fabricated using a PVA/KOH gel electrolyte which self-organizes into a well-defined three-dimensional porous structure that enhances moisture adsorption and transport. The humidity sensing mechanism is governed by changes in ionic conductance, which increases more than 70-fold as relative humidity rises from 10.89% to 81.75%, while maintaining a rapid and fully reversible response under ambient conditions. The device demonstrates excellent selectivity for humidity, with negligible sensitivity to temperature fluctuations from 0 to 95 °C and pressure variations between 0 and 6.8 kPa. The sensor is also successfully integrated into a non-contact switching system capable of detecting humidity gradients generated by an approaching fingertip, where its output shows a strong linear correlation with readings from a commercial reference instrument [56].

Bioinspired membrane-type humidity sensors face mechanical instability during cyclic swelling, hysteresis from trapped moisture, fabrication challenges in creating multiscale pores, environmental degradation, and lower electrical conductivity compared to inorganic sensors, which limits long-term performance. These biological inspirations reinforce the design logic of Trojan-type humidity sensors, in which confined interfacial phases and dynamic hydrogen-bond networks emulate the selective, efficient, and self-regulated ion and proton transport observed in natural systems such as membranes and ion channels [57].

Trojan-type humidity sensors are bioinspired because they replicate key functional attributes of biological moisture and ion transport systems. In cell membranes and ion channels, selective and efficient transport occurs within confined, hydrated nano-environments, where water acts as a critical mediator of rapid proton and ion movement through transient hydrogen-bond networks and proton relay processes. By emulating these structures and physicochemical processes, engineers can develop sensors with greater sensitivity, selectivity, and energy efficiency. These bioinspired approaches offer the potential for high performance using sustainable, abundant, and low-cost materials.

Plant-, animal-, and membrane-inspired humidity sensors collectively demonstrate the significant role of bioinspiration in advancing sensing technologies, each offering distinct advantages based on their natural counterparts. Plant-inspired designs enable efficient water transport and responsiveness through hierarchical and hygroscopic structures, while animal-inspired systems provide high sensitivity and adaptability by mimicking skin, hair, or other moisture-responsive biological surfaces. Membrane-based sensors, often fabricated from natural or biocompatible materials, deliver excellent flexibility, breathability, and real-time interaction with moisture. Among these, plant-based hygroscopic mechanisms are particularly promising for continuous humidity sensing because they provide fast, reversible responses driven by naturally occurring gradients in structure and composition.

These approaches support the development of highly sensitive, low-power, multifunctional devices, particularly well-suited for wearable and health-monitoring applications. However, challenges related to long-term stability, environmental robustness, and large-scale integration persist, underscoring the need for continued research to translate these bioinspired concepts into durable, versatile solutions for biomedical and environmental sensing.

## 3. Trojan Materials: Invisible Performance Enhancers

### Concept and Examples of Trojan Materials in Humidity Sensors

The Trojan materials strategy is based on the use of a functional disguise to facilitate entry into a protected system. Unlike direct intrusion methods, this technique utilizes the mimicry of natural or beneficial elements to gain host acceptance. By presenting an external structure recognized as safe, the agent can bypass defense mechanisms and infiltrate the system. Once internalized, they act as high-precision vehicles for delivering specialized payloads or performing site-specific tasks directly at the source. Within the scope of advanced science, such as nanomedicine and pharmacology, the Trojan material paradigm has evolved into a fundamental principle of structural nanomedicine, gene therapy, and biotechnology [57]. Its primary function lies in acting as an intelligent delivery vector, designed to mitigate the challenges of contemporary pharmacokinetics. This system facilitates the selective targeting of therapeutic agents to specific sites, preserving the integrity of the payload against systemic enzymatic degradation and premature immunological clearance. Beyond their applications in nanomedicine and pharmacology, Trojan materials have also been applied in physical and materials science, and electronics (e.g., [57,58,59,60,61]).

In the field of humidity sensors, several material systems reported in the literature can be interpreted as Trojan materials when analyzed through the lens of functional invisibility (e.g., [62,63]). Here, the term “Trojan material” does not refer to a specific chemical composition, but rather to the functional role and mode of integration of a material within a sensing matrix. Specifically, a component in the sensor is considered a Trojan material when it is embedded within the host matrix without significantly altering the device’s macroscopic morphology or structure. It acts as a hidden functional phase that enhances humidity-sensing performance in several ways. The Trojan material can serve as an adsorption enhancer, an energy-barrier modulator within the sensor matrix, and an interfacial mediator for ionic transport, rather than merely contributing typical bulk properties such as increased porosity or conductivity.

**Figure 2:**
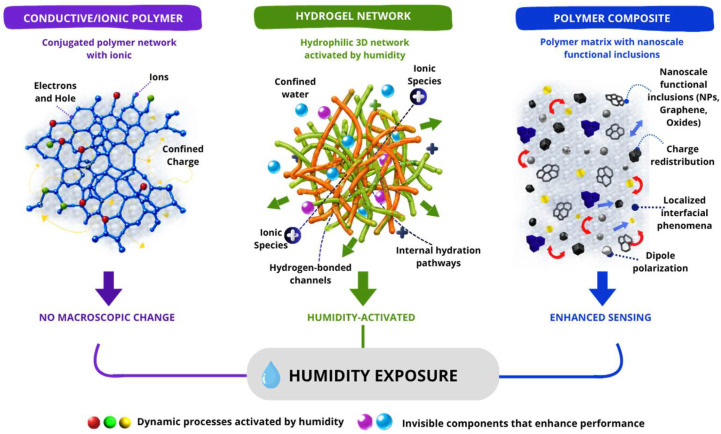
Trojan material effects in humidity-sensing polymers. Created using Canva Pro and Adobe Inc. (2025). Adobe Photoshop (Version 2025).

As an adsorption enhancer, the Trojan material is added to attract and retain water molecules at specific sites where they are most effective at generating an electrical signal, without necessarily increasing the material’s visible porosity [64,65]. As an energy-barrier modulator, the Trojan entity enhances the ease with which electrons hop from one site to another, thereby decreasing the activation energy required for electrical conduction [63,66,67]. In materials acting as interfacial mediators for ionic transport, these substances do not necessarily conduct charge themselves; instead, they create a “bridge” or environment that facilitates the movement of ions through the matrix [61,62]. In what follows, some examples of Trojan materials used in resistive and capacitive sensors are presented. A brief overview of their functional invisibility is provided, along with the key benefits for sensor performance (Table 1). Various Trojan materials can be identified in humidity sensor applications, including biopolymers, plant biomass-derived nanomaterials, metal oxides, metal–organic frameworks, and carbon-based materials (Figure 2).

In humidity sensors based on MOFs, MOFs function as localized adsorption enhancers as their large specific surface area and high porosity provide more active sites [64,65]. The pores act as the pathway for transporting water molecules [65]. Additionally, the adjustable structure of MOFs endows MOF-based humidity sensors with tunable hydrophilicity. While MOFs are often known for their low hydrophilicity, the introduction of hydrophilic ligands into the framework [70,71,72] or directly loading humidity-active materials into the MOFs [73,74] allows for an increase in their overall hydrophilicity.

In humidity sensors based on hybrid compound CNT yarns decorated with manganese oxide (MnO_2_), the process of energy-barrier modulation can be observed [67]. The CNTs provide a highly conductive electrical pathway, enabling rapid electron migration. The MnO_2_ offers active sites for the adsorption of H_2_O molecules. At the junction between the CNT and the metal oxide (the semiconductor), a Schottky junction (a potential barrier) is formed. When water molecules are adsorbed at this interface, they alter the charge density and flatten the energy barrier. This allows electrons to flow much more easily, resulting in a sudden increase in conductivity. When semiconductor metal oxides are combined with graphene or reduced graphene oxide (rGO), the energy-barrier modulation mechanism resembles that of CNTs but gains an additional physical dimension due to graphene’s two-dimensional (2D) structure. While CNTs provide one-dimensional transport, graphene offers a conducting sheet with a vast theoretical surface area. When metal semiconductors are anchored onto this sheet, the dominant mechanism becomes the formation of a 2D–3D heterojunction with ultra-sensitive, dynamic tuning of the Fermi level. When a metal semiconductor such as TiO_2_, Fe_2_O_3_, or ZnO [63,67] is deposited onto the graphene sheet, electron transfer occurs from the semiconductor to the graphene to align their Fermi levels. As water molecules adsorb onto the metal oxide surface and inject electrons into the semiconductor, a Schottky barrier forms along the entire two-dimensional interface, causing graphene to experience an electronic doping effect. In dry conditions, these barriers act as highly resistive energetic bottlenecks, keeping the system in a contained equilibrium. However, when ambient humidity changes, water molecules interact with the metal oxide, triggering a significant injection of electrons from within the structure. This electronic infiltration abruptly narrows and flattens the trans-interfacial potential barrier. Due to graphene’s atomic thickness, the two-dimensional sheet undergoes an instantaneous “chemical gating” effect, dynamically shifting its Fermi level. This geometric and electronic modulation, distributed across the entire planar surface, allows charge carriers to bypass energy fluctuations via quantum tunneling, causing the material’s resistance to collapse and resulting in an ultra-fast, linear, and highly sensitive macroscopic conductivity transition triggered by an otherwise invisible stimulus. In both cases, the Trojan-like behavior appears at the Schottky junction. For the CNT, the yarn seamlessly hosts the metal oxide nanostructures as benign, passive surface decorations; however, this hybrid integration quietly introduces millions of hidden electronic gates—the Schottky potential barriers—deep into the conductive matrix of the sensor. Similarly, for the metal oxide and graphene/rGO sheets, the sensor matrix incorporates both materials at the macroscale as structural components and passive conductors. Nevertheless, this union conceals a latent energetic trap that is the formation of many nanojunctions and hidden Schottky barriers throughout the entire volume of the material.

In humidity sensors where cellulose nanofibers (CNF) are incorporated into graphene oxide (GO)/cellulose nanofiber (GO/CNF) films to develop capacitive humidity sensors, CNFs serve as interfacial mediators for ionic transport and as localized adsorption enhancers [61]. GO and CNF form a heterostructure with channels and lamellar nanointerfaces. Introducing CNFs between GO sheets prevents the graphene from compacting or fully restacking. CNFs function as nanoscale spacers, creating an interconnected three-dimensional porous network of interfacial pathways. Water easily penetrates the interfaces formed between GO and CNF. The functional groups of both materials ionize the water, releasing protons (H^+^). This interfacial porous network serves as an ultra-fast mediator for the Grotthuss mechanism, allowing hydronium ions (H_3_O^+^) to hop and migrate along the interfaces with minimal friction, which increases the capacitance and dielectric response of the sensor to high levels. Both materials are highly hydrophilic, enhancing localized adsorption. Integrating hydroxyl-rich CNF into the GO framework increases the available active sites for water interaction through hydrogen bond formation compared to pristine GO membranes. GO contains epoxide and hydroxyl groups on its basal surface and carboxyl groups at its edges. In summary, introducing CNF into the GO matrix creates a synergistic effect: the CNFs act as localized adsorption enhancers by chemically attracting more water molecules into the film’s interior due to the abundance of -OH groups, and simultaneously open physical channels that serve as interfacial mediators for ionic transport, dramatically accelerating proton hopping (Grotthuss mechanism) and the dielectric polarization required for capacitive transduction. A Trojan effect occurs here because, at the macroscale, the sensor membrane incorporates cellulose solely as a structural support additive—a natural, innocuous, and biocompatible polymer—to provide flexibility to the film and prevent the restacking of the GO sheets. The system regards CNFs strictly as passive mechanical spacers. However, they covertly introduce latent hydrophilic active sites and interfacial channels into the material. In the presence of humidity, these channels activate from the inside out, triggering a chain ionization and significant dielectric polarization that sharply increases the sensor’s capacitance. Chitosan, a natural polymer used to develop resistive humidity sensors, serves also as an interfacial mediator for ionic transport and a localized adsorption enhancer [62]. It acts as a polymeric framework that is biomimetically insulating, passive, and stable. However, chitosan contains several functional groups, such as amines and hydroxyls, that form hydrogen bonds with environmental water molecules. When exposed to humidity, these functional groups open pathways in the matrix, promoting the autoionization of water and rapidly transforming the insulating polymer into a conductive electrolytic medium through the Grotthuss mechanism.

The examples presented as Trojan materials fundamentally differ from standard functional fillers, dopants, or nanocomposite reinforcements. Standard functional fillers superficially alter bulk electrical or porous properties, whereas atomic dopants intrinsically modify the host crystal lattice and electronic band gaps. On the other hand, nanocomposite reinforcements are introduced strictly to improve mechanical and thermal stability while remaining entirely passive during chemical transduction. Table 2 provides a comprehensive and comparative overview of the adsorption mechanism, energy barrier modulation, ionic transport mechanism, governing physical laws, and performance impacts that distinguish Trojan materials from standard material modifications.

## 4. Humidity Sensing Mechanisms

A comprehensive understanding of the physicochemical mechanisms underlying humidity sensing is fundamental to the rational design of high-performance sensing materials. Phenomena such as the Grotthuss proton-transfer mechanism in water, adsorption and desorption kinetics, proton transport, interfacial polarization, and humidity-dependent dielectric modulation act in concert to determine the macroscopic electrical response of polymeric and composite systems.

At low relative humidity (RH), the sensing behavior is predominantly governed by the physisorption of water molecules mediated by van der Waals forces and hydrogen bonding to the active sites of the material. With increasing RH, chemisorption processes and the formation of multilayer water films become increasingly important, giving rise to confined and/or interfacial water layers that can undergo partial dissociation to produce H^+^/H_3_O^+^ ionic species. The migration of these ions occurs primarily through proton hopping via the Grotthuss mechanism, which markedly enhances ionic diffusion and leads to a substantial increase in electrical conductivity at elevated RH levels [75]. The magnitude and nature of this effect depend greatly on the material type. In proton-conducting or polyelectrolyte polymers, protonic transport primarily determines the humidity response, whereas in composite systems such as carbon-nano-onion polymer nanocomposites, additional phenomena, including carrier compensation and humidity-induced polymer swelling, become more significant [76].

However, excessive swelling of amorphous regions at high relative humidity can disrupt long-range electronic percolation, progressively shifting the conduction mechanism toward ionic dominance. This behavior is observed in sulfonate-functionalized polythiophenes such as in PEDOT:PSS, where increased humidity induces microphase separation between hydrophobic (primarily electronic) and hydrophilic (ionic, water-swellable) regions. As water content increases, ionic conductivity rises markedly, while electronic transport is maintained through percolating π-stacked networks [77]. A comprehensive understanding of these mechanisms is crucial for accurately interpreting the performance of humidity sensors based on conductive polymers and hydrogels, as the underlying molecular processes directly determine the resulting electrical response. In such hydrated materials, proton transport proceeds predominantly via H^+^ migration along percolating networks of water molecules and/or polar functional groups embedded within the polymer matrix.

Kreuer describes two main pathways: (i) vehicular migration, in which protons travel with hydrating water molecules, and (ii) the Grotthuss mechanism, where successive hydrogen-bond exchanges and rapid water reorientation enable proton hopping between adjacent hydrogen-bonded molecules without large-scale molecular diffusion [78]. This enables efficient conduction in hydrated systems such as chitosan or alginate. In hydrogels and ionically conductive polymers, dynamic hydrogen-bond networks form continuous channels for proton migration, with their density and mobility strongly dependent on moisture content and the balance of dissociated ions [79,80]. This is characteristic of Trojan-type systems, where confined charges act as hidden agents controlling conductivity. Dielectric modulation arises from interfacial polarization (Maxwell–Wagner–Sillars effect) in composites: humidity-induced permittivity contrasts cause charge accumulation at interfaces, altering the sensor’s dielectric response [81]. In mixed ionic–electronic polymers, this polarization is amplified by reversible redox doping, which locally tunes energy barriers and enables self-regulated conductivity [82].

Water-assisted ionic conduction integrates these effects: water uptake increases the dielectric constant, lowers resistance, and creates additional ionic pathways. The coupling of water adsorption, polarization, and proton transport underlies the high sensitivity and stability of hydrogel- and hydrated polymer-based humidity sensors [83].

Overall, Trojan materials exhibit a predominantly water-driven response due to water’s high dielectric constant, strong hydrogen-bonding capability, and exceptional proton mobility associated with the Grotthuss mechanism [84,85]. However, concerns about the selectivity of the system must be evaluated. Polar non-aqueous species, such as alcohols and certain volatile organic compounds, can interact with the polymer matrix through hydrogen bonding or dipole–dipole interactions, causing local variations in dielectric permittivity and ionic conductivity [86]. However, the response elicited by these analytes is typically weaker and slower than that produced by water, due to their reduced capacity to promote ionic dissociation and sustain extended proton-conducting hydrogen-bond networks. Consequently, although some cross-sensitivity to polar non-aqueous molecules cannot be completely excluded, the overall transduction behavior is dominated by water adsorption, resulting in a favorable selectivity profile for humidity sensing applications.

## 5. Classes of Bioinspired Self-Sensing Materials with Trojan Materials

### 5.1. Conductive and Ionic Polymers

Conductive and ionic polymers (CPs) are among the most extensively studied material systems for humidity sensing in various environments because of their inherent responsiveness to water molecules and compatibility with scalable deposition methods such as spin coating, dip coating, and printing [39,87,88]. In the context of conductive or ionic polymers, we interpret the Trojan entities as the mobile charge carriers (electrons, holes, or ions) confined within the polymer framework. These charge carriers act as hidden functional agents that regulate interfacial charge transport, polarization processes, and energy-barrier dynamics under varying humidity conditions, thereby enhancing sensing performance without causing detectable changes in the macroscopic morphology of the material [24,80].

The moisture-sensing response of conductive and ionic polymers arises from the combined effects of water adsorption, proton migration, dielectric modulation, interfacial polarization, and the structural characteristics of the polymer matrix [89]. At low RH levels, water molecules are first adsorbed into polar functional groups, such as hydroxyl, sulfonic, and amine groups, through hydrogen bonds and weak intermolecular interactions, forming a tightly bound primary hydration layer [23,75,90]. Under these conditions, the adsorbed water remains localized and changes in conductivity are limited, as continuous charge transport pathways have not yet formed. As RH increases, greater water absorption occurs via physisorption, leading to the development of multilayered and interconnected water domains within the polymer structure [75]. The partial dissociation of these confined water regions generates mobile ionic species, including H^+^ and H^3^O^+^, which increase conductivity via proton hopping through the Grotthuss mechanism, along with the diffusion of carrier ions [23,75,90]. The gradual transition from adsorption-dominated behavior under low-humidity conditions to efficient ionic transport under high-humidity conditions determines key detection characteristics, including sensitivity, hysteresis, response and recovery times, and long-term operational stability [90,91,92].

Beyond adsorption phenomena, the moisture response of conductive polymers depends strongly on morphology, crystallinity, doping level, phase segregation, and conductive percolation effects [84,88]. Amorphous and porous domains generally favor water diffusion and adsorption due to their larger free volume and enhanced chain mobility [84], whereas highly crystalline regions restrict swelling and improve mechanical robustness [84]. Increased crystallinity therefore tends to enhance environmental stability and reduce signal drift, although often at the expense of lower sensitivity and slower adsorption kinetics [25,84,90]. Similarly, conductive polymers operating near the percolation threshold may exhibit very high humidity sensitivity because relatively small structural variations can induce large conductivity changes. However, these systems are also more susceptible to hysteresis and instability under cyclic hydration–dehydration conditions due to progressive disruption of conductive pathways [25].

CPs, particularly PANI, PEDOT:PSS, and polypyrrole (PPy), have attracted considerable attention due to their π-conjugated structures and humidity-dependent electronic transport properties [93]. In these materials, water adsorption modulates the protonation state, doping level, and interchain charge transport, resulting in measurable changes in resistance or impedance. Generally, conductive polymer systems offer relatively fast electronic responses, easy integration into resistive sensing platforms, and compatibility with low-temperature fabrication [84]. However, their performance is often limited by swelling-induced drift [94], hydrolysis [95], dedoping processes [96], and reduced long-term environmental stability under prolonged humid exposure [88,97].

PANI-based humidity sensors are among the most widely studied conductive polymer systems because of their multiple oxidation states and reversible protonation-deprotonation behavior. These materials typically show a decrease in resistance with increasing relative humidity, as water adsorption enhances proton-assisted hopping between localized states and increases charge carrier density [39,98]. Imali et al. demonstrated that PANI-based resistive humidity sensors operate over a wide humidity range of 0–97% RH, exhibiting a linear impedance response with high correlation (R^2^ = 0.99) and a sensitivity of approximately 1.1701 Ω/%RH [98]. The devices also showed relatively low hysteresis (about 2.1%) and good repeatability over multiple cycles. For dynamic performance, response times ranged from approximately 150 to 220 s, while recovery times reached up to 150 s, depending on humidity conditions. Similarly, Ragazzini et al. developed cellulose/PANI composite sensors in which humidity detection is governed by water adsorption and proton-assisted electron hopping within the polymer network, resulting in increased conductivity as RH rises [39]. Their devices exhibited a linear response in the 30–50% RH range, with a sensitivity of approximately 13%, good reproducibility, and response/recovery times ranging from 90–370 s and 87–490 s, respectively. Although PANI-based sensors offer high sensitivity, simple fabrication, and wide operating ranges, prolonged exposure to humidity can induce desorption, hydrolysis, and microstructural rearrangements, which may progressively compromise electrical reproducibility and long-term stability [99].

PEDOT:PSS exhibits intrinsically high conductivity due to its phase-separated morphology, in which PEDOT-rich conductive domains are dispersed within a hydrophilic PSS matrix [100]. In these mixed ionic-electronic conductors, the main PEDOT structure, with conjugated π bonds, supports electronic transport, while the hydrated and structurally disordered PSS-rich regions facilitate ionic migration [101]. Water absorption causes swelling of the hydrophilic PSS phase and microstructural rearrangement, which can enhance ionic conductivity and dielectric response while preserving electronic transport through permeable PEDOT pathways [100]. Bornemann et al. reported that this behavior improves connectivity between PEDOT domains and increases charge carrier mobility, resulting in reproducible detection responses over a wide range of humidity, typically between 20% and 80% RH, demonstrating the suitability of PEDOT:PSS for outdoor humidity detection applications [100].

Silva et al. investigated PEDOT:PSS blended with hygroscopic polyethylene oxide (PEO), where moisture detection is governed primarily by water absorption in the PEO phase [102]. The absorbed moisture causes the polymer matrix to swell and increases the separation between the conductive PEDOT domains, resulting in pronounced increases in electrical resistance under humid conditions. These resistive sensors exhibited a wide detection range of approximately 6–92% RH, as well as good repeatability, stability, and the ability to detect rapid changes in humidity [102]. Although PEDOT:PSS-based sensors generally show stable and reproducible performance at moderate humidity levels, excessive swelling at high RH can progressively disrupt long-range electronic percolation pathways, shifting the transport mechanism toward ionic conduction and potentially increasing hysteresis and signal drift [77].

PPy and related conductive polymers display structure-property relationships similar to those of other intrinsically conductive polymers, although charge transport is generally dominated by local hopping mechanisms [93]. In these materials, humidity sensitivity mainly results from water-assisted modulation of the π-conjugated structure and proton-assisted charge transfer through hydrogen-bond networks, which facilitate changes in conductivity under humid conditions [84]. Ravikiran et al. described PANI and PPy thin-film humidity sensors that operate over a wide range of 10–97% RH, synthesized via in situ polymerization (PANI) and chemical oxidative polymerization (PPy), followed by deposition as thin films on substrates [92]. The PPy sensor exhibited sensitivities of up to 71%, along with response and recovery times of approximately 60 s, demonstrating good linearity and long-term stability [92].

Hybrid polymer systems incorporating PPy into porous or hydrophilic matrices have also shown improved detection behavior due to optimized water adsorption kinetics and greater accessibility of water molecules to the conductive network [84]. For example, polystyrene (PS)/PPy composite films exhibited rapid and reproducible responses to water vapor, confirming the effectiveness of combining PPy with hydrophilic matrices to enhance sensor performance [89]. Despite their wide moisture detection ranges, relatively fast response dynamics, and high sensitivity, repeated swelling and deswelling cycles can progressively disrupt the conductive pathways in PPy-based systems, reducing long-term reproducibility and environmental robustness [89]. Although these conductive polymer systems rely primarily on electron transport modulated by moisture-induced structural effects and doping processes, there is also a distinct class of moisture-sensitive materials that operate predominantly via ionic conduction mechanisms.

Unlike conductive polymers, ionic polymer systems function primarily through water-assisted ion transport rather than electronic conduction. Representative materials include polyvinyl alcohol (PVA), polyionic liquids (PILs), and zwitterionic polymers [55,91,103]. In these systems, absorbed water promotes ionic dissociation and facilitates proton mobility in the hydrated regions of the polymer matrix, leading to significant changes in conductivity or impedance as RH increases [91]. At higher humidity levels, interconnected water networks formed by hydrogen bonds further enhance ionic transport, resulting in high humidity sensitivity commonly reflected in impedance spectra and response-recovery behavior [90].

Compared to conductive polymer systems, ionic polymers generally exhibit higher sensitivity at high relative humidity. However, extensive water absorption can also lead to swelling, slow recovery dynamics, ion leaching, and long-term signal drift. Panwar et al. developed a PVA-based ionic sensing platform by incorporating sugarcane extract into the polymer matrix, then molding and thermally drying it to form an ionic membrane with silver electrodes [104]. The sugarcane extract introduced natural ionic species, sulfur- and iron-containing compounds, and fibrous structures from bagasse, which enhanced ionic conductivity, dielectric properties, and interaction with water within the hydrated polymer network [104]. Similarly, Yu et al. reported cross-linked polyionic liquid humidity sensors that demonstrated high sensitivity, low hysteresis (<5% RH), and stable cyclic operation due to controlled hydration dynamics and efficient ionic conduction pathways [91]. In a subsequent study, the incorporation of zwitterionic functionalities further improved adsorption–desorption reversibility and operational stability by stabilizing local hydration structures [103]. However, excessive water absorption can still cause hydrolytic degradation and mechanical fatigue during prolonged cyclic exposure.

Conductive and ionic polymers are attractive materials for moisture sensing because of their tunable physicochemical properties, mechanical flexibility, and compatibility with low-temperature, scalable fabrication techniques [91,93]. Although their detection behavior depends on different transport processes, the performance of both systems is strongly influenced by water–material interactions and changes in the polymer’s microstructure under humid conditions [88]. Highly hydrophilic materials generally provide greater sensitivity, while more ordered or semi-crystalline systems typically offer better environmental stability and mechanical durability. Therefore, future developments should focus on balancing sensitivity, response speed, and long-term stability through careful control of polymer morphology, phase organization, and transport pathways [23].

### 5.2. Polymer Composites

Polymer-based composite materials have emerged as highly effective platforms for humidity sensing due to their tunable physicochemical properties and the synergistic interactions between polymer matrices and functional nanostructures [105]. As summarized in Table 3, a wide variety of composites-incorporating semiconductor metal oxides (SMOs), carbon-based materials and ceramics, have been explored in resistive, capacitive, and impedance sensor configurations. These nanostructures, including nanoparticles, nanorods, nanosheets, and tubular morphologies, play a crucial role in enhancing sensor performance by increasing surface area [87,106] porosity [87,107], and the density of active sites available for water adsorption [40,107]. Polymer-based materials and composites are widely used in humidity sensing because they support water absorption, flexibility, hygroscopicity, and high surface area, which favor the Grotthuss mechanism in moisture transport [23,108].

To better understand their sensing behavior, the dominant sensing mechanism in most composites involves water adsorption through chemisorption at low RH and physisorption at higher RH. At low RH, chemisorbed water forms tightly bound hydroxyl groups, enabling limited electronic or proton transport. As humidity increases, multilayer physisorption forms hydrogen-bonded water networks, facilitating proton hopping (Grotthuss mechanism) and significantly increasing ionic conductivity [108,110,125]. In polymer-carbon nanocomposites, additional effects such as humidity-induced swelling [117,126] and modulation of conductive percolation pathways [117,118] further contribute to the sensing response.

**Figure 3:**
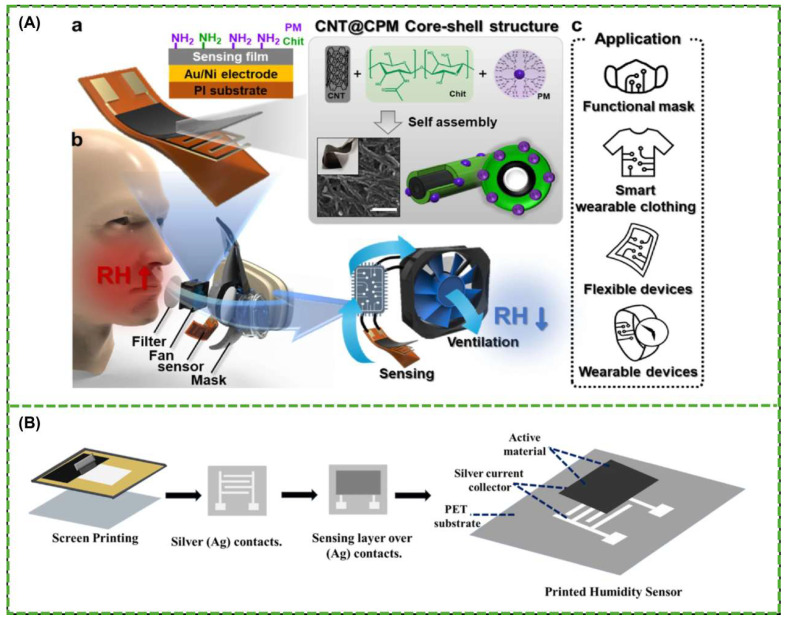
(**A**) Schematic illustration of the fabrication and application of a flexible humidity sensor based on a CNT@CPM nanocomposite (CNT core with Chit/PM shell) immobilized on Au/Ni electrodes over a flexible PI substrate: (**a**) sensor fabrication process; (**b**) schematic representation of a respiration mask with an integrated ventilation system; and (**c**) examples of wearable smart devices (reproduced with permission [118]. Copyright 2022, Springer Nature Link). (**B**) Schematic illustration of the fabrication process of a humidity sensor on a PET substrate, including screen printing of silver electrodes, deposition of the sensing layer, and final device assembly (reproduced with permission [87]. Copyright 2026, Springer Nature Link).

Carbon-based materials, such as CNTs [118] graphene [117], and its derivatives-namely GO [106,127] and rGO [128] have been widely used in humidity sensors as dopants for reinforcing polymer matrices. Their outstanding electrical conductivity, large specific surface area, tunable surface chemistry, and ability to form highly responsive percolation networks make them especially attractive for this application [129]. As a result, polymer-carbon nanocomposites often show enhanced humidity sensing performance, including higher sensitivity [117,126,128], faster response and recovery times [108,130] and improved stability [106,117]. Compared with many conventional polymer-only humidity sensors reported in the literature, which often exhibit response/recovery times ranging from tens of seconds to several minutes together with lower resistance variation at high RH levels, these nanocomposites can achieve response/recovery times below 20 s and sensing responses exceeding 90%, highlighting their competitive performance relative to current state-of-the-art flexible humidity sensors. For instance, Chethan et al. [106] developed an ultra-sensitive humidity sensor based on a PANI/GO composite, exhibiting a high sensing response (≈93%), fast response/recovery times (4 and 7 s, respectively), low detection limits, negligible hysteresis, high sensitivity, and excellent stability. These response and recovery times are considerably lower than those commonly reported for conventional polymer-based humidity sensors, demonstrating the effectiveness of GO incorporation in accelerating water adsorption/desorption kinetics and proton transport mechanisms. A CNT-based flexible humidity sensor with a core–shell CNT@CPM structure (using chitosan and PAMAM) was developed by Kim et al. [118] Figure 3A, exhibiting high sensitivity, response/recovery times below 20 s, low hysteresis (−0.29 to 0.30% RH), and outstanding mechanical durability (over 15,000 bending cycles), enabling reliable long-term performance and real-time respiratory monitoring in smart wearable applications. Such mechanical robustness exceeds that of many flexible humidity sensors reported in the literature, where performance degradation is frequently observed after only a few thousand bending cycles, reinforcing the suitability of this platform for wearable electronics and long-term monitoring applications. In another study, it was demonstrated that bacterial cellulose/GO composite films, prepared via a simple and low-cost dry film-forming method and subsequently reduced with L-ascorbic acid to enhance electrical conductivity, exhibit high humidity sensing performance, with a resistance change of up to ≈94%, fast response/recovery times (13 and 47 s, respectively), and effective noncontact detection of breathing patterns, highlighting their potential for health monitoring applications [116].

SMOs have also been extensively studied as fillers in polymer-based humidity sensors due to their chemical inertness, low cost, portability, ease of processing, and high humidity sensing performance across a wide temperature range [130]. Common examples include TiO_2_ [40,107,131], ZnO [110,111,112], CuO [87,112], tin oxide (SnO_2_) [114,132,133], and nickel oxide (NiO) [115] which are typically incorporated as nanoparticles or nanorods. Their combination with polymers enhances humidity sensing by increasing water adsorption, modifying charge transport pathways, and strengthening polymer–water interactions. Compared with pristine polymer sensors, SMO-polymer composites generally exhibit broader operating humidity ranges, improved sensitivity, and enhanced linearity due to the higher density of active adsorption sites and improved interfacial charge transfer processes. A ZnO-coated PMMA microfiber humidity sensor fabricated using direct drawing and sol–gel methods has been reported, where ZnO coatings significantly improve performance. Sensitivity increases from 0.1191 dBm/% (uncoated) to 0.1791 dBm/% for ZnO nanostructures and 0.2159 dBm/% for ZnO nanorods. This nearly twofold increase in sensitivity compared with the pristine PMMA sensor highlights the strong contribution of ZnO nanostructures to water adsorption and signal transduction efficiency. The device also offers improved sensitivity, low-cost fabrication, and suitability for compact humidity sensing applications [111]. In another study, Manjunatha et al. [87] (Figure 3B) reported a flexible resistive humidity sensor fabricated on a polyethylene terephthalate (PET) substrate using screen-printed PPy/CuO nanocomposite inks, where the incorporation of CuO enhanced hydrophilicity and sensing performance, with the optimal 1:1 composite exhibiting high sensitivity over a wide humidity range (22–97% RH), fast response and recovery times (≈50 and 60 s, respectively), excellent stability, and negligible hysteresis. The broad operating humidity range and low hysteresis indicate reliable sensor performance under fluctuating environmental conditions, which remains a significant challenge for many resistive humidity sensing platforms. Recently, Devesa et al. [40] developed a sustainable, cost-effective resistive humidity sensor using a casting method, by incorporating TiO_2_ nanoparticles into cellulose extracted from potato peels, to improve water adsorption and sensing performance, resulting in enhanced sensitivity compared to pristine cellulose, as confirmed by humidity-dependent impedance measurements.

Overall, combining polymers with functional nanomaterials enables the design of high-performance humidity sensors with tailored properties and enhanced performance, making these composites promising candidates for applications ranging from environmental monitoring to wearable electronics and healthcare diagnostics. Several of these systems already approach state-of-the-art performance, although challenges related to long-term stability, hysteresis, and scalable fabrication remain.

Different classes of polymer-based humidity sensors present clear performance trade-offs. Polymer-only sensors are simple and low-cost but generally show lower sensitivity and slower response. Polymer–carbon nanocomposites improve sensitivity and dynamics due to enhanced conductivity and percolation effects but may suffer from stability and drift issues. SMO-based composites provide better chemical stability and broader humidity range, although often with slower response and reduced flexibility. More recently, Trojan-type hybrid architectures have shown the best overall sensing performance by combining high adsorption capacity with efficient charge transport, but their fabrication is more complex and less scalable. Overall, the optimal material choice depends on balancing sensitivity, stability, cost, and scalability for the target application.

Hybrid materials that combine Trojan-type architecture with polymer-based composites are increasingly used in humidity sensing applications because of their high sensitivity and tunable physicochemical properties. In these systems, the polymer matrix acts as a mechanically flexible and water-permeable scaffold, while the Trojan-type components provide enhanced and, in some cases, selective adsorption sites for water vapor Humidity-induced changes in the electrical conductivity or dielectric permittivity of the composite can be converted into quantifiable signals, enabling precise detection and monitoring of environmental humidity fluctuations. Additionally, molecular-level structural and chemical tailoring of both the polymer phase and the Trojan-type domains enables sensors with improved operational stability, faster response and recovery times, and adjustable performance characteristics, making them particularly suitable for flexible electronics and advanced environmental monitoring platforms.

### 5.3. Hydrogel- and Water-Compatible Systems

Nature frequently exploits water-rich matrices as functional camouflage environments, allowing materials to operate while remaining undetected or non-disruptive to surrounding systems. In this context, hydrogels and water-compatible systems are among the most compelling classes of bioinspired self-sensing materials for advanced humidity sensing as Trojan materials. Their high-water content, structural similarity to biological tissues, and intrinsic responsiveness to environmental humidity make them especially well-suited for this role.

One of the most crucial functions of humidity sensors is accurately monitoring moisture levels in surrounding environments, both indoors and outdoors. However, conventional humidity sensors are often limited by poor mechanical flexibility, low optical transparency, and relatively complex or time-consuming fabrication processes. In contrast, hydrogel-based humidity sensors offer enhanced stretchability, transparency, and adaptability, making them highly attractive for next-generation applications, especially in flexible integrated technologies.

The performance of hydrogel-based systems is supported in their ability to dynamically interact with water molecules through absorption, diffusion, and desorption mechanisms. Structurally, hydrogels are three-dimensional porous polymeric networks formed via physical or chemical crosslinking of hydrophilic components, enabling them to retain significant amounts of water [134]. Their tunable physicochemical features allow them to act as “invisible enablers”, embedding sensing functionalities within a soft and hydrated matrix. As a result, hydrogel-based humidity sensors have found applications across diverse fields, including electronic skin, respiratory monitoring, environmental sensing [134], and more recently, smart window technologies, where their optical transparency and environmental adaptability are particularly advantageous [135].

Although wearable applications have been the primary focus of research, broader opportunities remain underexplored. For instance, precise humidity monitoring is essential in museums and libraries for the preservation of artifacts, paintings, and historical documents. Similarly, industrial sectors such as semiconductor manufacturing, textiles, and food processing require reliable humidity control to ensure product quality and process stability [136]. These contexts highlight the versatility and growing relevance of hydrogel-based sensing platforms beyond conventional use cases.

Recent advances in hydrogel-based humidity sensors have been driven by the rational design of material compositions and architectures that enable efficient transduction of humidity variations into measurable signals. These signals may arise from changes in volume, optical properties (e.g., color variation), or, more commonly, electrical parameters such as resistance, impedance, and capacitance [90]. As previously stated, the use of polymer blends and composite systems has proven particularly effective in enhancing sensing performance, as combining different materials allows fine-tuning of sensitivity, response time, and stability [108]. Furthermore, chemical modification strategies, including grafting, copolymerization, crosslinking and the formation of interpenetrating polymer networks have been extensively explored to achieve an optimal balance between hydrophilicity and hydrophobicity, which is critical for precise and reliable humidity sensing.

Commonly used polymeric materials, particularly hydrogels, are widely employed in humidity sensor fabrication due to their ease of processing, cost-effectiveness, and compatibility with established manufacturing techniques [10,137]. Their sensing performance is closely linked to their swelling behavior, which depends on factors such as synthesis methods, water uptake capacity, release dynamics, and structural stability. Hydrophilic functional groups within the polymer backbone facilitate water absorption, while crosslinking ensures structural integrity by preventing dissolution and enabling reversible responses to humidity changes. Natural polymers such as chitosan, cellulose, sodium alginate, and silk fibroin are rich in hydrophilic groups, making them especially attractive for use in humidity sensors.

PVA is a widely used polymer in humidity sensing due to its hydrophilicity, biocompatibility, and favorable mechanical and chemical stability [138]. Its porous structure and high density of hydrophilic functional groups enable efficient moisture absorption, leading to measurable changes in electrical properties. Although intrinsically a poor electrical conductor, PVA can become conductive when combined with other polymers or conductive fillers, allowing the sensing function to be embedded within the bulk matrix. In this sense, PVA-based systems act as adaptable hosts, concealing conductive pathways, and sensing mechanisms within a transparent and flexible structure. Its swelling behavior and high moisture expansion coefficient further contribute to its sensitivity.

Recent studies demonstrate how polymer blending strategies further enhance this Trojan functionality. A notable example involves a ternary PVA/gelatin/chitin polymer blend, fabricated as flexible films via solution casting [119]. In this system, moisture uptake enhances ionic charge transport, resulting in a humidity-dependent resistive response over a moderate RH range (≈11–84%). Each component contributes synergistically: gelatin enhances water uptake, chitin improves conductivity and structural stability, and PVA provides mechanical integrity. The ternary blend demonstrates good sensitivity across varying humidity levels, with a good response time (≈15 min). In addition to sensing, the material displays antibacterial properties, illustrating how multiple functionalities can coexist within a single, seemingly simple matrix.

Chitosan-based hydrogels have long been employed in medical, pharmaceutical and environmental applications [139] and more recently emerged as promising platforms for humidity sensing. Within the framework of Trojan materials, chitosan-based systems exemplify how naturally derived polymers can act as functionally invisible matrices, embedding sensing capabilities within soft, hydrated environments that mimic biological tissues. Their strong affinity for water molecules, arising from abundant hydroxyl and amine functional groups, enables efficient hydrogen bonding and proton transport [139] allowing humidity detection to occur intrinsically within the material without the need for exposed or rigid sensing elements.

**Figure 4:**
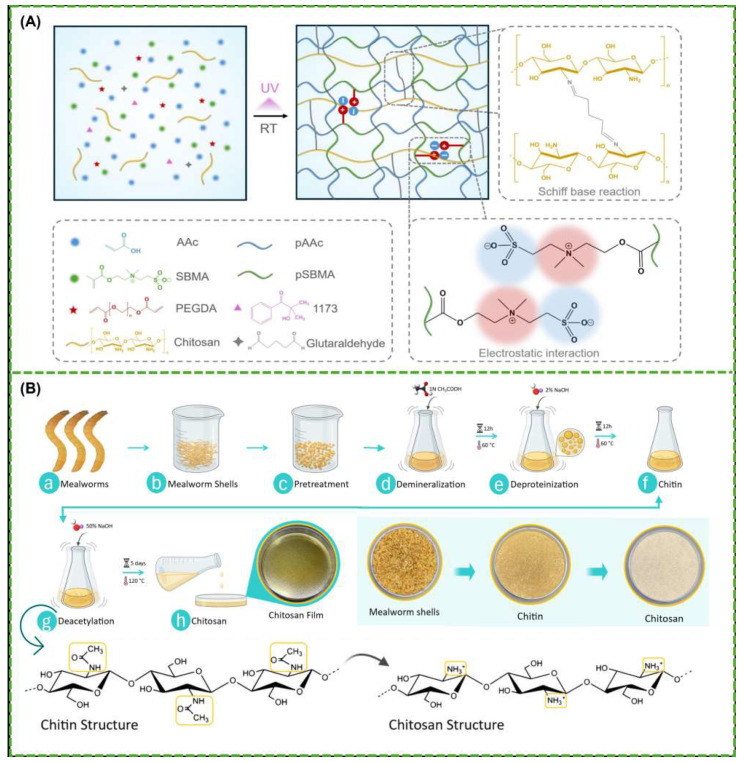
(**A**) Design of CS-pSBMA/pAAc hydrogel coatings (reproduced with permission [134]. Copyright 2025, Sensors, MDPI). (**B**) Schematic representation of chitosan extraction from mealworm shells and subsequent film preparation: (a) mealworms; (b) shells; (c) pulverization; (d) demineralization using acetic acid; (e) deproteinization via alkaline treatment (NaOH); (f) chitin isolation; (g) deacetylation of chitin to obtain chitosan; and (h) casting into films. Photographs of the intermediate and final products are shown above (reproduced with permission [135]. Copyright 2026, Chemical Engineering Journal, Elsevier).

Nettey-Oppong et al. highlight the development of flexible and sustainable humidity sensors based on chitosan/PVA composites [120], Figure 4A. In this study, biomass-derived chitosan extracted from Tenebrio molitor larvae was combined with PVA and integrated with copper interdigitated electrodes to form a resistive sensor. The sensing mechanism relies on water-induced proton conduction through hydrogen-bond networks, achieving a broad sensing range (6–97% RH) with fast response (18.22 s) and recovery (22.39 s) times. The reversible hydrogen bonding capability of chitosan plays a central role in this performance, concealing the active sensing processes (from a Trojan materials perspective), while maintaining environmental adaptability. Although the hygroscopic nature of the fabricated sensor enhances its sensitivity to atmospheric moisture, prolonged exposure may compromise its structural integrity and long-term stability, leading to gradual degradation. Nevertheless, the use of bio-waste-derived precursors further reinforces the sustainability and low-impact nature of this approach, while IoT integration demonstrates its applicability in real-time environmental monitoring, smart agriculture and industrial process control.

The Trojan paradigm is even more evident in multifunctional hydrogel systems designed to operate in complex environments. For instance, Wei et al. reported an anti-biofouling, humidity-responsive hydrogel coating based on chitosan reinforced with a zwitterionic polymer network composed of poly(sulfobetaine methacrylate) (pSBMA) and poly(acrylic acid) (pAAc) [121], Figure 4B. The incorporation of sulfobetaine groups significantly enhances water uptake and provides strong resistance to biological contamination, enabling stable operation in bioactive and marine environments. In this case, the material not only performs humidity sensing but also passively resists external interference, effectively “hiding” its functionality behind antifouling behavior. The sensor exhibits high sensitivity, good linearity, and reliable repeatability, delivering a humidity-dependent output voltage that increases from 16 V to 30 V as the RH rises from 28% to 80%. As expected for hydrophilic materials that absorb large amounts of water, excessive swelling and mechanical degradation may occur. The chitosan-pSBMA/pAAc hydrogel exhibited a synergistic degradation mechanism attributed to its interpenetrating polymer network (IPN) structure, resulting in a more controlled and stable degradation process rather than sudden structural collapse. Overall, this materials-level strategy integrates passive anti-biofouling with humidity-enhanced charge transfer, offering a promising pathway for reliable triboelectric nanogenerator (TENGs) operation in biomedical and marine environments.

Cellulose-based materials further illustrate how natural polymers can be engineered into Trojan-like sensing systems. As a widely available and biocompatible polysaccharide, cellulose, and particularly nanocellulose, offers a high density of hydroxyl groups that facilitate strong interactions with water molecules. This enables rapid adsorption even at low humidity levels, resulting in high sensitivity. The sensing mechanism is typically governed by swelling-induced structural changes, which modulate conductive pathways and electrical properties through reversible hydrogen bonding [140]. Importantly, these processes occur within a seemingly inert matrix, allowing the material to function as an embedded sensor without altering its external form or appearance.

A representative example is the successful fabrication of a carboxymethyl cellulose (CMC) hydrogel membrane for humidity sensing reported by Pinming et al., achieved using a spin-coating technique [122]. The membrane was chemically cross-linked with epichlorohydrin, forming a stable hydrogel layer that functions as the active moisture-absorbing component in a resistive-type humidity sensor. The sensing performance was evaluated by measuring the electrical resistance of the CMC hydrogel membrane at different relative humidity levels (RH = 53%, 75%, 84%, and 93%). The results demonstrate that the CMC-based resistive humidity sensor operates effectively within this high-humidity range, exhibiting a response time on the order of approximately 100 s.

The concept extends to integrated environmental systems, such as multifunctional hydrogels designed for greenhouse applications, enabling simultaneous functions as an anti-fogging coating and a humidity-responsive sensing platform. This hydrogel is composed of poly(N-acryloyl glycinamide) (PNAGA) and poly(acrylamide) (PAAm) as the primary long-chain polymer networks, forming a robust and highly hydrophilic matrix [123]. Furthermore, glycerol and sodium chloride are incorporated into the system to enhance environmental adaptability, enabling the hydrogel to maintain performance over a wide range of temperatures and humidity levels. The additives enhance environmental stability and introduce ionic conductivity, enabling resistance-based humidity sensing through internal structural and ion-transport changes. In this context, the sensing performance is evaluated by monitoring the relative change in resistance as a function of RH, providing a quantitative measure of the hydrogel’s sensitivity to environmental moisture variations.

A study by Syrový et al. demonstrated the potential of CNFs, derived from bagasse fibers, for the fabrication of humidity sensors [124]. In this work, CNF-based films were investigated both with and without the incorporation of poly(ethylene glycol) (PEG) as a plasticizer, allowing the authors to assess its influence on material performance. The addition of PEG improved the ductility and flexibility of the films and led to noticeable changes in sensor kinetics, highlighting the role of the plasticizer in modulating moisture diffusion and response dynamics. Carbon-based interdigitated electrodes (IDE) were directly printed onto the CNF substrates to form the sensing devices. The resulting CNF-based humidity sensors exhibited a pronounced impedance response, with changes reaching up to four orders of magnitude as the RH increased from 20% to 90%, demonstrating high sensitivity to environmental moisture variations. Despite this high sensitivity, the material retains the appearance and properties of a simple cellulose film, effectively concealing its sensing capability within a natural, biodegradable matrix.

Overall, these examples demonstrate how hydrogel- and water-compatible systems embody the principles of nature-inspired Trojan materials. Owing to their rapid response and recovery [136] hydrogel matrices are particularly well suited for real-time humidity monitoring. As previously discussed, their performance can be further enhanced through material engineering strategies such as polymer blending, controlled crosslinking and the incorporation of plasticizers, which enable fine-tuning of sensitivity, stability and mechanical properties. By embedding sensing functionalities within hydrated, bio-sourced matrices, these materials enable advanced humidity sensing while preserving structural integrity and environmental compatibility. This ability to operate “invisibly” while delivering high performance positions them as key components in the next generation of adaptive, multifunctional sensing technologies. A remaining challenge is that pure polymers often exhibit poor mechanical robustness, structural instability, and prolonged degradation behavior, which can compromise long-term stability and adversely affect sensor performance. In addition, water uptake may induce further effects, including the leaching of charged species and the migration of plasticizers and ions across different regions of the material. Therefore, future studies should focus on overcoming these limitations by evaluating long-term performance degradation and developing strategies to enhance the mechanical properties and overall stability of the sensor.

Table 3 provides a summary of recently reported representative examples of humidity sensing systems with Trojan-like functionalities and presents an overview of various humidity sensors based on different material categories. The details of each material category were discussed in the respective sections.

### 5.4. Advantages and Limitations of Trojan Materials

The advantages of Trojan materials in humidity sensing systems go beyond isolated performance improvements, indicating a broader shift in the design and integration of sensing functionalities. As previously discussed, these materials enable the incorporation of active components within a host matrix without significantly altering its external geometry, flexibility, or mechanical integrity, thus establishing a paradigm of non-invasive functional enhancement [1,2,3,4].

Within this framework, the primary benefit of Trojan materials is their ability to intensify interactions between water molecules and the sensing medium. By introducing additional adsorption sites, facilitating moisture diffusion, and enhancing charge transport pathways, they strengthen the coupling between microstructure and electrical response, which is widely recognized as governing sensing behavior in advanced polymeric and composite systems [5,6,7,141]. Consequently, signal transduction becomes more efficient, particularly under low-humidity conditions and across wide operating ranges. However, the same water-mediated interactions responsible for enhanced sensitivity may also induce excessive swelling, hysteresis, and signal drift under prolonged outdoor humid exposure. Particularly in conductive and ionic polymer systems, repeated adsorption and desorption cycles can progressively disrupt conductive pathways, alter charge transport mechanisms and compromise long-term reproducibility and environmental stability.

This design strategy closely reflects principles found in biological systems, where multifunctionality arises from hierarchical organization and efficient resource use. As discussed in Section 2, nature-inspired materials use structural complexity and adaptive interfaces to optimize water management and responsiveness [16,23,24]. Trojan materials apply these biological concepts to engineered systems, enabling improved performance without structural redesign or increased material complexity. However, reproducing biologically inspired adaptive behavior in synthetic systems remains challenging because continuous moisture exposure can accelerate physicochemical aging, hydrolysis, and degradation processes that adversely affect operational reliability over extended periods.

Another major advantage of Trojan materials is the ability to modulate internal properties independently of the macroscopic structure. By embedding functional phases that selectively tune hydrophilicity, conductivity, or dielectric behavior, Trojan materials allow precise control over sensing mechanisms while preserving the overall form factor of the device [6,8,93]. This decoupling of function and morphology is particularly beneficial for developing flexible, lightweight, and conformable humidity sensors. However, extensive water uptake may also promote ion migration, dedoping phenomena, and leaching of charged species, especially in conductive and ionic polymer systems, leading to slow recovery dynamics, increased hysteresis and long-term signal instability.

In addition, the Trojan material strategy inherently supports multifunctionality. As highlighted in previous sections, especially for conductive polymers, composites, and hydrogel-based systems, embedded additives can simultaneously enhance water adsorption, stabilize the material structure, and promote ionic or electronic transport [5,6,7,72]. Conductive polymers such as PEDOT:PSS and PPy provide relatively fast electronic responses, compatibility with low-temperature fabrication methods, and straightforward integration into resistive sensing platforms. However, prolonged exposure to high relative humidity can cause swelling, hydrolysis, and progressive disruption of conductive percolation pathways, reducing environmental robustness and long-term reproducibility. Ionic polymers generally exhibit higher sensitivity at elevated humidity levels due to enhanced ion mobility, although excessive water absorption may lead to slow recovery, ion leaching, and signal drift.

Hydrogel- and water-compatible systems represent another important class of Trojan-inspired materials due to their high-water content, structural similarity to biological tissues, and intrinsic responsiveness to environmental humidity. Their exceptional flexibility, stretchability, and optical transparency make them highly attractive for next-generation wearable and flexible sensing technologies. However, despite their high sensitivity and adaptability, hydrogel-based systems often exhibit the slowest response and recovery dynamics among Trojan-inspired humidity sensing materials because water diffusion and desorption processes within the three-dimensional polymeric network are intrinsically slower. Moreover, prolonged exposure to humid environments may compromise structural integrity and long-term stability, leading to gradual degradation of the hydrogel network. Pure polymeric hydrogel systems often exhibit limited mechanical robustness, structural instability, and progressive degradation, while extensive water uptake can cause ion migration, plasticizer diffusion, and leaching of charged species, further compromising sensor durability and operational consistency. Finally, Trojan materials contribute to the advancement of sustainable and scalable humidity sensing technologies. Their compatibility with bio-based materials, such as cellulose and chitosan, and their ability to enhance performance without complex fabrication processes align with the growing demand for environmentally responsible solutions [5,6,7,72]. Additionally, their compatibility with flexible electronics and Internet of Things (IoT)-enabled platforms positions Trojan-inspired systems as promising candidates for bridging the gap between laboratory-scale developments and real-world humidity sensing applications. However, challenges related to large-scale reproducibility, homogeneous dispersion of embedded functional phases, and long-term environmental stability must be addressed before widespread commercialization can be achieved.

Overall, integrating Trojan materials with bioinspired design has accelerated the development of next-generation humidity sensors based on conductive polymers, ionic systems, composites, and hydrogels. These materials collectively demonstrate how biological concepts can inspire engineering solutions that deliver enhanced sensitivity, flexibility, multifunctionality, and adaptability. However, practical implementation remains constrained by swelling-induced instability, hysteresis, environmental degradation, and durability limitations during prolonged operation. Therefore, future research should focus on improving mechanical reinforcement, controlling water-induced physicochemical changes, optimizing interfacial compatibility, and systematically evaluating long-term operational reliability under realistic environmental conditions to fully realize the potential of Trojan-inspired humidity sensing technologies.

## 6. Benchmark Humidity Sensors

Nowadays there are several commercial solutions for humidity sensors, supporting applications from industrial process control to environmental monitoring. Accurate and reliable sensing is crucial for providing controlled conditions and enabling early detection of degradation phenomena such as corrosion in real-world environments. Commercial sensors have evolved considerably, driven by growing demands for environmental sustainability, system efficiency, and seamless integration into smart and distributed platforms. These sensors use various physical and chemical transduction mechanisms, including capacitive, resistive, and optical principles, and employ organic, inorganic, or hybrid materials. Manufacturers are increasingly incorporating nanostructured and functional materials to enhance sensitivity, response time, and long-term stability, ensuring robust performance under diverse environmental conditions [88,142]. Continuous advancements have focused on improving key performance parameters, including sensitivity, linearity, hysteresis, response and recovery times, and long-term stability, while also enabling miniaturization, flexibility, and compatibility with integrated electronic and wearable platforms [10].

From a structural perspective, humidity sensors typically consist of a hygroscopic sensing layer deposited onto a substrate, coupled with electrodes that enable signal transduction. Depending on the operating principle, these devices are broadly classified into capacitive, resistive, thermal, piezoelectric (surface acoustic wave, SAW), and optical sensors, each offering distinct transduction pathways and material requirements [84]. Here, particular emphasis is placed on electrical transduction-based sensors.

Capacitive humidity sensors are the most widely used technology, accounting for most of the commercial hygrometer markets. Their operation is based on changes in the dielectric constant of a hygroscopic material upon water adsorption, typically polymeric films such as polyimide. These sensors have been commercially available since the 1980s and 1990s, with continuous improvements driven by integration with microelectronic systems. Modern devices, such as the Sensirion SHT series [143], Bosch BME280 [144], Honeywell HIH series [145], Vaisala HUMICAP^®^ and DRYCAP^®^ [146], and Rotronic HygroClip probes [147] incorporate on-chip signal conditioning and temperature compensation, enabling direct digital or analog output and seamless integration into IoT platforms. These commercial capacitive sensors typically exhibit accuracies in the range of ±1 to ±3% RH, with high-end devices achieving ±0.5% RH, while covering a full operating range of 0–100% RH. Response times are generally below 10 s, often reaching 1–8 s depending on airflow conditions, and hysteresis is typically below 1–3% RH for high-quality devices. They operate across a broad temperature range, commonly from −40 °C to +80 °C, with advanced platforms such as the Sensirion SHT3x and SHT4x series extending up to +125 °C, while long-term drift is usually below 0.25–0.5% RH per year.

Among these, the Sensirion SHT3x series is a widely used platform, offering variants such as SHT30, SHT31, and SHT35, with increasing levels of performance (approximately ±2% RH, ±1.5% RH, and ±1% RH accuracy, respectively). These sensors are based on CMOSens^®^ technology, which integrates a capacitive humidity-sensing element with a temperature sensor and signal-processing circuitry on a single chip [148].

In this configuration, interdigitated electrodes combined with a hygroscopic polymer layer form a capacitor whose dielectric properties change as a function of ambient humidity. This variation is measured electrically and converted into a calibrated digital signal, enabling accurate and stable humidity determination. Additional features such as I^2^C communication, adjustable sensor addressing, programmable alerts, heater functionality for condensation mitigation, and a wide supply voltage range (2.15–5.5 V) further facilitate integration into microcontroller-based systems Datasheet SHT3x-DIS. Similarly, devices such as the Bosch BME280 are optimized for low power consumption (typically ≈3.6 µA in standard operation) and compact design, making them particularly suitable for wearable and mobile applications, while maintaining acceptable accuracy (±3% RH), fast response, and good long-term stability [149].

Resistive humidity sensors function by detecting changes in electrical resistance resulting from ionic conduction within hygroscopic materials as they absorb water. Available since the 1970s and 1980s, these sensors remain widely used in low-cost and consumer applications because of their simple fabrication, high sensitivity, and ease of integration into electronic systems. Representative commercial devices include the DHT11 [145], DHT22/AM2302 [150], and AM2301 sensors [151] which integrate humidity and temperature sensing elements with onboard signal processing to provide calibrated digital outputs. The DHT11 typically provides an accuracy of ±5% RH over a limited range of 20–80% RH, while the DHT22/AM2302 improves performance to ±2–3% RH across 0–100% RH, with response times in the range of 2–10 s depending on conditions [145,150]. Additional commercially available sensors include the HR202 resistive sensor, which operates as an analog variable resistor with typical response times of ≈10–30 s [152], and proprietary polymer-based solutions such as the Hument HPR series, which employ engineered sensing membranes for enhanced sensitivity and stability.

Assessing these commercial technologies through the lens of Technology Readiness Levels (TRL) highlights the wide maturity gap within the humidity sensing landscape. Capacitive sensors based on polyimide and CMOS-compatible fabrication, such as the Sensirion SHT series and Vaisala HUMICAP^®^, have reached TRL 9, representing fully deployed and field-proven systems. Resistive sensors such as the DHT22 and ceramic-based platforms similarly occupy TRL 8–9 for standard applications. In contrast, emerging platforms including graphene oxide-based sensors, bioinspired nanostructured layers, and flexible cellulose composites remain at TRL 3–5, confined to laboratory validation without demonstrated manufacturability or field reliability. Bridging this gap is the central challenge for the next generation of humidity sensing technologies.

Ionic polymers such as poly(2-acrylamido-2-methylpropane sulfonic acid) and PEO exhibit high sensitivity because of increased ionic mobility when water is absorbed. In commercial sensors like the DHT11 and DHT22 families, the sensing element is typically an organic macromolecular film combined with a temperature sensor (such as an NTC thermistor) for compensation [153].

Carbon-based nanomaterials, including graphene, GO, and multi-walled carbon nanotubes (MWCNTs), are increasingly studied for their high surface area and rapid response [154]. Notably, these material classes have reached commercial maturity, with companies such as TDK Corporation and Figaro Engineering Inc. developing and commercializing ceramic-based sensing devices, particularly for gas and humidity detection [155,156]. Inorganic salts such as LiCl remain common in traditional resistive sensors because their electrical resistance strongly depends on moisture content. Composite systems, including MWCNT/polyimide and SnO_2_/rGO hybrids, have been developed to combine the high sensitivity of nanomaterials with improved mechanical stability [157,158]. Structurally, these sensing materials are typically deposited onto interdigitated electrodes, often fabricated from noble metals such as gold or platinum. As humidity increases, water molecules are absorbed and dissociated within the sensing layer, leading to an increase in ionic charge carriers and a corresponding decrease in electrical resistance. This mechanism underpins the high sensitivity observed in resistive sensors but also contributes to their susceptibility to environmental influences.

## 7. Challenges and Future Directions

Despite advances, several key challenges still limit the large-scale use of humidity sensors for outdoor monitoring. Electric sensors, such as capacitive and resistive types, offer several advantages. However, in the specific case of capacitive sensors, they are affected by environmental factors such as condensation, ultraviolet exposure, and chemical contaminants, which can cause temporary measurement errors drift, and long-term material degradation. To mitigate these effects, both material selection and device architecture are critical. The sensing layer must have high hygroscopicity, chemical and mechanical stability, and a linear dielectric response to humidity variations. In most commercial applications, the dielectric material is a hygroscopic polymer [159] with polyimide being the most widely used due to its excellent thermal stability, chemical resistance, and broad operating temperature range [159]. Other commonly used materials include cellulose derivatives such as cellulose acetate butyrate, which are particularly suitable for flexible and printed electronics [160] and polymethylmethacrylate (PMMA), often employed in low-cost sensing platforms due to its favorable film-forming properties [156].

Long-term stability, especially regarding drift, remains a critical parameter for humidity sensors. Standard polymer-based sensors typically show drift values of about 0.5–1% RH per year, while optimized polyimide-based systems can reduce this to below 0.25–0.5% RH per year. For high-reliability and industrial applications, inorganic materials such as aluminum oxide and mesoporous silica are often used, as they exhibit significantly lower drift (typically less than 0.25% RH per year) due to their resistance to swelling and aging [161]. Emerging electro-ceramic materials, such as calcium–magnesium–iron–titanium oxides, further demonstrate excellent stability under harsh environmental conditions [162]. Additionally, advanced protective coatings and engineered polymer layers are increasingly implemented in commercial sensors to minimize the effects of contaminants such as dust, oils, and chemical vapors, thereby enhancing long-term performance and reliability [163]. In the specific case of resistive humidity sensors, they have several inherent limitations, including pronounced hysteresis (often 3–6% RH), signal drift (commonly 1–2% RH per year or higher under harsh conditions), and limited long-term stability due to material degradation, particularly under high humidity or condensation. Polymer-based sensing layers are especially susceptible to swelling, dissolution, and aging, which leads to a gradual loss of sensitivity. In contrast, ceramic-based sensors offer superior stability, chemical resistance, and durability, making them more suitable for harsh environments, although typically at higher cost and with reduced flexibility [84,157].

Additional challenges include strong temperature dependence, which requires compensation strategies, and susceptibility to contamination by dust, oils, and chemical vapors that may permanently alter sensor response. Saturation effects at high humidity levels can cause slow recovery times and performance degradation. To address these limitations, modern sensor designs increasingly incorporate protective coatings and hybrid material systems that combine polymer sensitivity with ceramic robustness [164,165]. Recent advances in nanomaterials, including graphene oxide, carbon nitride, and metal oxides, have significantly improved response times and sensitivity, while flexible and biodegradable materials such as cellulose/polyaniline composites enable emerging applications in wearable and sustainable sensing platforms [158].

In addition to the previous challenges, there are more general issues that should be considered. A primary issue is the scalable, reproducible fabrication of advanced sensing materials, especially nanostructured and bioinspired architectures. Although these materials offer high sensitivity and fast response, scaling from laboratory to mass production is difficult and often results in performance variability. Cost-effective manufacturing is another constraint: dense sensor networks over large areas require low-cost devices, yet combining affordability with durability and high performance, particularly for advanced materials and complex architecture, remains challenging. Long-term environmental robustness is also problematic. Outdoor sensors face fluctuating temperatures, humidity cycles, UV radiation, pollutants, and biological fouling, which can degrade sensing layers, cause signal drift, and reduce reliability. Stable long-term operation requires robust materials, improved encapsulation, and smarter calibration. Power consumption is another limiting factor, especially for remote or autonomous systems. While IoT integration enables real-time monitoring, it also requires highly efficient energy use to prevent frequent maintenance or battery replacement. Finally, achieving high selectivity and reducing cross-sensitivity to temperature and volatile compounds remain technical hurdles. Addressing these issues is crucial for accurate, reliable humidity measurements in complex outdoor environments.

Outdoor humidity sensors are expected to become more integrated, adaptive, and energy-efficient. Future systems will use multifunctional platforms that measure humidity, temperature, pressure, and air quality, supporting richer datasets and improved environmental modelling. Closer integration with the IoT will enable real-time data collection, remote analysis, and large-scale deployment in smart agriculture, climate monitoring, and urban infrastructure. Advances in materials science, especially bioinspired materials, will increase sensitivity, reduce power consumption, and enhance water–surface interactions. Self-regenerating sensing layers could extend device lifespans and reduce maintenance in harsh conditions. Combined with low-power electronics and energy harvesting, these advances will enable autonomous, long-lasting sensors for continuous operation in remote or resource-limited environments.

Future research in outdoor humidity sensing should prioritize transitioning from high-performance prototypes to scalable, field-deployable technologies. This requires developing fabrication strategies that ensure reproducibility and cost efficiency without compromising sensitivity or response time. Special attention should be given to manufacturing techniques compatible with large-area and flexible substrates to enable widespread deployment in distributed monitoring networks. Improving long-term environmental stability remains essential, with efforts focused on robust encapsulation methods, anti-fouling surfaces, and adaptive calibration strategies to mitigate signal drift during prolonged exposure to harsh outdoor conditions. Another important direction is advancing material innovation, particularly through the design of bioinspired and nanostructured sensing layers that enhance water adsorption dynamics while maintaining ultra-low power operation. These materials can be engineered to provide rapid response, high selectivity, and resilience to environmental stressors. Additionally, integrating humidity sensors into interconnected systems based on the IoT enables real-time data processing, predictive analytics, and intelligent decision-making across environmental monitoring platforms.

## 8. Conclusions

Humidity sensors are now essential components in outdoor environmental monitoring, supporting applications ranging from climate observation to precision agriculture and smart infrastructure. This review article demonstrates that, at the core of humidity sensor evolution, inspiration drawn from plants, animals, and biological membranes is being used to design high-performance platforms for environmental humidity monitoring. By mimicking nature’s efficient water-management architectures, researchers can, at least in part, overcome the inherent limitations of traditional sensors. Combining these bio-inspired designs with the functional dimensions of Trojan materials (materials that activate in response to environmental stimuli) creates a paradigm shift in sensor development. Recent advances in nanostructured and bioinspired materials have significantly improved sensor sensitivity, response time, and miniaturization, enabling more accurate and distributed sensing. At the same time, integrating these devices into interconnected systems based on the IoT has expanded their functionality, allowing real-time data acquisition, remote monitoring, and data-driven decision-making.

Despite this progress, challenges with scalability, cost-effective manufacturing, long-term environmental stability, and energy efficiency continue to limit widespread deployment in outdoor settings. Addressing these limitations requires a multidisciplinary approach that combines materials science, device engineering, data processing, and system integration.

Trojan material architectures in which confined charge carriers or interfacial domains dictate humidity sensing while preserving macroscopic morphology represent a highly versatile platform for developing next-generation intelligent sensors. Promising research directions include hierarchically engineered polymer–hydrogel networks, iono-electronic hybrid composites, and biodegradable frameworks that provide both high functional performance and enhanced environmental sustainability [79,80,83]. Elucidating the atomistic mechanisms of charge transport, the long-term stability of interfacial regions, and the coupling between ionic and electronic conduction pathways remains a critical open challenge [81,82]. Meanwhile, emerging technological opportunities are expanding this field toward AI-assisted sensor design and optimization, where data-driven models establish quantitative correlations between structure, composition, and humidity response [23]; self-powered sensing platforms that integrate piezoelectric or triboelectric nanogenerators [166,167]; and multifunctional electronic skins and neuromorphic systems that leverage the adaptive, self-regulated charge dynamics intrinsic to Trojan materials [166,167]. Advances in biodegradable polymers and environmentally benign fabrication strategies are also expected to enable sustainable, transient, or recyclable devices capable of autonomous operation in flexible and wearable form factors [25]. Collectively, these perspectives position Trojan materials as a conceptual and technological bridge between conventional sensing media and intelligent, energy-autonomous soft electronic systems.

## Figures and Tables

**Table 1:** Examples of Trojan materials in resistive and capacitive humidity sensors, their role and key benefits.

Trojan Material	Sensor Type	Mechanism of Enhancement	Key Benefits	Ref.
Chitosan	Resistive	Adsorption enhancer; Interfacial mediators for ionic transport	Fast adsorption and desorption rates; High sensitivity; High reproducibility; Biocompatible; Nontoxicity; Excellent reversibility;	[62,68]
Graphene oxide/cellulose nanofiber	Capacitive	Interfacial mediators for ionic transport Adsorption enhancer	Fast response/recovery time; Low hysteresis; High sensitivity	[61]
ZnO/Graphene	Resistive	Energy-barrier modulation	Fast response and recovery times; Low hysteresis; High sensitivity	[63]
TiO_2_/Graphene	Resistive	Energy-barrier modulation	High sensitivity High Stability Cost-effectiveness	[67]
NH_2_-Mil-125(Ti)/Si-NPA (MOFs composites)	Capacitive	Adsorption enhancer	High sensitivity; Low hysteresis; High reproducibility; Low response and recovery times	[69]
CNTs/MnO_2_	Resistive	Energy-barrier modulation	High stability; High sensitivity; Fast response and recovery times; Flexible sensor	[66]

**Table 2:** Theoretical framework contrasting the multifunctional mechanisms of Trojan materials against standard functional fillers, dopants, and nanocomposite reinforcements in humidity sensors.

Theoretical Dimension	Standard Functional Filler	Dopant Modifier	Nanocomposite Reinforcement	Trojan Material (Unified Approach)
Adsorption Mechanism	Passive and strictly surface-bound; Limited to the exposed external surface area	Alters local chemical hydrophilicity but yields no gain in effective surface area	Acts as an inert or physical barrier; Reduces humidity diffusion pathways	Adsorption Enhancer: Creates anti-aggregation 3D templates or tailored microporous channels for active capillary condensation
Energy Barrier Modulation	Creates parallel conductive pathways without altering the intrinsic matrix junctions	Statically alters the electronic band structure by creating lattice defects/oxygen vacancies	Entirely neutral toward the electronic energy profile	Barrier Modulator: Dynamically regulates band bending at the heterojunction via interfacial field effects upon H_2_O exposure
Ionic Transport Kinetics	Tortuous and discontinuous charge conduction across poor particle-to-particle interfaces	Negligible contribution; Strictly focused on the host lattice’s intrinsic electronic conduction	Blocks ionic pathways, severely increasing mass transfer resistance	Interfacial Mediator: Provides continuous, hydrophilic nanostructured highways that optimize the Grotthuss mechanism in confined spaces
Governing Physical Law	Classical Percolation Theory	Kröger–Vink Defect Thermodynamics/Crystal Field Theory	Continuum Mechanics/Rule of Mixtures	Interfacial Field Effect; Quantum Confinement; Grotthuss Proton Diffusion
Impact on sensor performance	Non-linear response; Baseline drift; Premature saturation	May improve baseline conductivity but risks distorting or collapsing the active phase	Enhances structural and mechanical robustness; Passive toward signal transduction	Ultra-high sensitivity; Rapid response/recovery kinetics; Minimized hysteresis

**Table 3:** Representative examples of humidity sensing systems with Trojan-like functionalities, classified by material type (conductive/ionic, composites, and hydrogels), including their composition, enhancement mechanisms, and key performance indicators.

Material System	Sensor Type	Components	Enhancement Mechanism	Performance Indicators	Ref.
Conductive/Ionic Systems					
PEO/PVA	Resistive	PEO/PVA polymer	PEO enhances ionic conductivity, while PVA improves water adsorption and thermal stability, enabling a temperature-independent response	RH range: 10–60% Sensitivity: High Response time: 9 s Recovery time: 16 s Hysteresis: 12.5%RH Applications: Environmental monitoring	[55]
Polyaniline (PANI)	Impedance	PANI	Humidity-induced protonation and hydrogen bonding in PANI increase ionic conductivity and reduce impedance	RH range: 0–97% Sensitivity: 1.1701 Ω/%RH Response time: ≈150–220 s Recovery time: ≈150 s Hysteresis: ≈2.1%RH Applications: Environmental sensing	[98]
PEDOT:PSS	Resistive	PEDOT:PSS	Humidity-induced PSS swelling increases PEDOT spacing, reducing charge transport and increasing resistance	RH range: 20–80% Sensitivity: ≈480% ∆R Applications: Structural health monitoring	[100]
PS/PPy	Resistive	PPy on electrospun PS	High-surface-area electrospun fibres with a PPy conductive coating improve water adsorption and proton conduction	RH range: 11–97% Sensitivity: ≈128.6% Response time: ≈55 s Recovery time: ≈77 s Applications: Environmental monitoring	[89]
PIL film	Impedance	Cross-linked PIL (ionic polymer)	Ionic groups enhance adsorption and dissociation, promoting ionic conduction	RH range: 5–35% Sensitivity: 48.0 (5–35%RH) Response time: ≈1 s Recovery time: ≈10 s Hysteresis: 0.2%RH Applications: Low humidity monitoring	[91]
Zwitterionic PIL film	Impedance	Zwitterionic polymers (SB-type)	Zwitterionic groups regulate adsorption/desorption, enhancing ionic conduction and fast recovery	RH range: 11–95% Sensitivity: 4862.8 Response time: ≈1 s Recovery time: ≈15 s Hysteresis: 0.6%RH Applications: Humidity sensing (fast-response systems)	[103]
Composite Systems					
TiO_2_ nanoparticles-based paste (water, propylene glycol, n-propanol, HPC, Solsperse 40000)	Resistive	TiO_2_ nanoparticles-based paste	Increases active sites for water adsorption and diffusion	RH range: 5–70% Response time: 40 s–3 min Recovery time: 50 s Application: environmental monitoring	[107]
Potato peel cellulose/TiO_2_	Resistive	TiO_2_ nanoparticles incorporated into potato peel cellulose	Improves adsorption and conduction	Response time: almost linear in the range 0–100% Sensitivity: 241 for RH = 100% Application: low-cost humidity sensing	[40]
PPy/ TiO_2_	Resistive	TiO_2_ nanoparticles and PPY	Facilitate water vapor adsorption and increases conductivity	RH range: 11 –97% Response time: 75 s Recovery time: 98 s Sensitivity: 94%	[109]
PLA/PANI–ZnO	Resistive	electrospun fibers	Enhances water adsorption and conductivity	RH range: 20–90% Response time: 85 s Recovery time: 120 s Hysteresis: ≈4.2% at low RH and ≈8.9% at 70% RH Application: humidity sensing	[110]
PMMA/ZnO	optical	PMMA microfiber coated with ZnO	The transmission of the microfiber coated with ZnO nanorods coating decreases linearly with the increase in humidity	RH range: 50–80% Sensitivity: 0.2159 dBm/% Linearity: more than 98% Resolution: 1.7 Application: humidity sensing	[111]
PVP/ WO_3_/ZnO	Resistive	electrospun fibers	Creating additional paths for charge transport increases conductivity	RH range: 11–97% Hysteresis: ≈7% Sensitivity: high Response time: 9 s Recovery time: 1 s Application: testing fruit freshness, healthcare product moisture control, and multiple industrial applications	[112]
PPy/CuO	Resistive	PPy/CuO nanocomposite conductive ink	Enhances surface area and porosity; creates more adsorption sites for water molecules	RH range: 22–97% Hysteresis: negligible Response time: 50 s Recovery time: 60 s Application: humidity sensing	[87]
PEO/CuO/MWCNTs	Capacitive	PEO/CuO/MWCNTs: 3% composite nanofibers	Increases dielectric constant and active sites	RH range: 30–90% Sensitivity: 53,837.6% Response time: 20 s Recovery time: 11 s Application: monitoring of health and medical facilities, environmental measurements, engineering instruments and remote control of various electronic devices	[113]
PVA/SnO_2_	Resistive	Multilayer structure (alumina substrate, interdigitated silver electrodes, PVA–SnO_2_ layer)	Adds hydrophilic adsorption sites	RH range: 50–90% Sensitivity: 53,837.6% Response time: 167 s Recovery time: 559 s Application: humidity sensing	[114]
PANi/NiO	Resistive	NiO nanoparticles incorporated into PANi matrix	Increases water absorption and conductivity	RH range: 5–90 Sensitivity: 7.929 kΩ/RH Response time: 60 s Recovery time: 90 s Application: humidity sensing	[115]
PANi/GO	Resistive	Interdigitated electrodes + PANI–GO composite film	Provides functional groups provides high surface area; enhances water adsorption; facilitates ion transport	RH range: 7–97% Sensitivity: 93.4% Hysteresis: negligible Response time: 4 s Recovery time: 7 s Application: ultra-sensitive sensing	[106]
Bacterial cellulose/partially rGO	Resistive	Composite film	Promotes hydrogen-bond network for proton transport	RH range: 0–100% Hysteresis: low Response time: ≈13 s Recovery time: ≈47 s Application: human health monitoring and noncontact sensing	[116]
Water-borne polyurethane/hydroxyethyl cellulose/xanthan gum/glycerol/graphene	Resistive	PET substrate + Interdigitated chromium /gold electrodes + graphene polymer composite sensing film	Facilitate water adsorption	RH range: 35%, 57%, and 79% Sensitivity: ≈ 1123% Response time: 61.1 s Recovery time: 92 s Application: respiratory detection and contactless gesture monitoring	[117]
PM-embedded Chit/CNT	Resistive	PI substrate + Au/Ni electrode + sensing film (PM-embedded Chit/CNT)	Provides a highly conductive pathway	RH range: 30–100% Sensitivity: 56.7–111.1% Hysteresis: very low (−0.29 to 0.30% RH) Response time: 10–40 s Recovery time: 10–40 s Application: human healthcare monitoring system	[118]
Hydrogel Systems					
PVA/gelatin/chitin	Resistive	Ternary polymer blend	Moisture uptake enhances ionic charge transport	RH range: 11–84% Response time: 15 min	[119]
Chitosan/PVA	Resistive	Laminate polymer composite	Water-induced proton conduction through hydrogen-bonds	RH range: 6–97% Sensitivity: 2.43 kΩ/%RH Response time: 18.22 s Recovery time: 22.39 s Applications: environmental monitoring, smart agriculture and industrial process control	[120]
Chitosan-pSBMA/pAAc	Triboelectric	Interpenetrating polymer network	Materials-level approach	RH range: 28–80% Applications: marine environments	[121]
CMC cross-linked with epichlorohydrin	Resistive	Cross-linked polymer	Cross-linking	RH range: 53%, 75%, 84% and 93% Response time: 100 s	[122]
PNAGA/PAAm with glycerol and sodium chloride	Resistive	Crosslinked long-chain polymers	Enrichment with free ions and glycerol to introduce ionic conductivity	Applications: intelligent greenhouse and agricultural films	[123]
CNF/PEG	Impedance	Biocomposite film	Plasticizer improves ductility and flexibility of the films	RH range: 20–90% Response time: 200/265 (with PEG) s Recovery time: 1020/490 (with PEG) s Applications: packaging	[124]

Abbreviations: Chit—chitosan; CMC—Carboxymethyl cellulose; CNF—Cellulose nanofibrils; CNTs—carbon nanotubes; CuO—copper oxide; GO—graphene oxide; MWCNTs—Multi-walled Carbon Nanotubes; NiO—nickel oxide; pAAc—Poly(acrylic acid); PAAm—poly(acrylamide); PANI—polyaniline; PEDOT:PSS Poly(3,4ethylenedioxythiophene):poly(styrenesulfonate); PS—Polystyrene; PPy—Polypyrrole; PIL—Poly(ionic liquid) PEG—Poly(ethylene glycol); PEO—Polyethylene oxide; PIL—Poly(ionic liquid); PLA—Polylactic acid; PM—PAMAM dendrimer ethylenediamine core, generation 3; PMMA—polymethylmethacrylate; PNAGA—Poly(N-acryloyl glycinamide); PPy—polypyrrole; PVA—Polyvinyl alcohol; PVP—Polyvinylpyrrolidone; RH—Relative humidity; rGO—reduced graphene oxide; SCN—Sugarcane extract; SnO_2_—tin oxide; TiO_2_—titanium dioxide; WO_3_—Tungsten oxide; ZnO—zinc oxide.

## Data Availability

No new data were created or analyzed in this study. Data sharing is not applicable to this article.
